# Negative Regulation of Bone Formation by the Transmembrane Wnt Antagonist Kremen-2

**DOI:** 10.1371/journal.pone.0010309

**Published:** 2010-04-27

**Authors:** Jochen Schulze, Sebastian Seitz, Hiroaki Saito, Michael Schneebauer, Robert P. Marshall, Anke Baranowsky, Bjoern Busse, Arndt F. Schilling, Felix W. Friedrich, Joachim Albers, Alexander S. Spiro, Jozef Zustin, Thomas Streichert, Kristina Ellwanger, Christof Niehrs, Michael Amling, Roland Baron, Thorsten Schinke

**Affiliations:** 1 Institute of Osteology and Biomechanics, University Medical Center Hamburg Eppendorf, Hamburg, Germany; 2 Harvard School of Dental Medicine and Harvard Medical School, Boston, Massachusetts, United States of America; 3 Institute of Pathology, University Medical Center Hamburg Eppendorf, Hamburg, Germany; 4 Department of Clinical Chemistry, University Medical Center Hamburg Eppendorf, Hamburg, Germany; 5 Division of Molecular Embryology, Research Program Cell and Tumor Biology of the German Cancer Research Center and the Center for Molecular Biology of the University of Heidelberg (DKFZ-ZMBH) Alliance, German Cancer Research Center, Heidelberg, Germany; University of Western Ontario, Canada

## Abstract

Wnt signalling is a key pathway controlling bone formation in mice and humans. One of the regulators of this pathway is Dkk1, which antagonizes Wnt signalling through the formation of a ternary complex with the transmembrane receptors Krm1/2 and Lrp5/6, thereby blocking the induction of Wnt signalling by the latter ones. Here we show that *Kremen-2* (*Krm2*) is predominantly expressed in bone, and that its osteoblast-specific over-expression in transgenic mice (*Col1a1-Krm2*) results in severe osteoporosis. Histomorphometric analysis revealed that osteoblast maturation and bone formation are disturbed in *Col1a1-Krm2* mice, whereas bone resorption is increased. In line with these findings, primary osteoblasts derived from *Col1a1-Krm2* mice display a cell-autonomous differentiation defect, impaired canonical Wnt signalling and decreased production of the osteoclast inhibitory factor Opg. To determine whether the observed effects of Krm2 on bone remodeling are physiologically relevant, we analyzed the skeletal phenotype of 24 weeks old *Krm2*-deficient mice and observed high bone mass caused by a more than three-fold increase in bone formation. Taken together, these data identify Krm2 as a regulator of bone remodeling and raise the possibility that antagonizing KRM2 might prove beneficial in patients with bone loss disorders.

## Introduction

Bone is constantly remodelled through the activities of bone-forming osteoblasts and bone-resorbing osteoclasts [1], [2]. A relative increase of bone resorption over bone formation can result in osteoporosis, one of the most prevalent diseases in the aged population [3], [4]. It is therefore of hallmark clinical importance to identify molecules specifically regulating the differentiation and activity of osteoblasts, since these can potentially serve as targets for osteoanabolic therapy. Moreover, as these molecules should be accessible to drugs, they should ideally be located in the extracellular space or at the cell surface, for instance as a receptor for a given ligand. In this regard, the identification of the secreted molecule SOST and the transmembrane protein LRP5 as regulators of bone formation in humans was a major breakthrough [5]–[9].

LRP5, together with LRP6, is the human orthologue of the *Drosophila* protein arrow, which serves as a co-receptor for wingless, the fly homologue of mammalian Wnt ligands [10]. Inactivating mutations of the human *LRP5* gene result in osteoporosis pseudoglioma syndrome, and a similar phenotype has been observed in mice with a targeted deletion of *Lrp5* [5], [11]. Likewise, activating mutations of Lrp5 in mice and humans cause osteosclerosis, a high bone mass disorder resulting from increased osteoblast activity [6], [7], [12]. In addition, several investigators have reported that specific single nucleotide polymorphisms within the *LRP5* gene are associated with decreased bone mineral density and increased risk of osteoporotic fractures [13]–[15]. Based on this cumulative evidence, but also due to its transmembrane localization, LRP5 has been considered an excellent target molecule for osteoanabolic therapy.

A second key regulator of bone formation in humans is the secreted protein SOST, which is specifically produced by osteocytes and acts as a negative regulator of osteblast activity [16], [17]. As it was the case for *LRP5*, the importance of the *SOST* gene for bone mass was first uncovered by human genetics, where it has been found that the loss of SOST expression or function causes either van Buchem disease or sclerostosis, two related high bone mass conditions caused by excessive bone formation [8], [9]. Likewise, while *Sost*-deficient mice displayed osteosclerosis, an osteoblast-specific over-expression of *Sost* resulted in an opposite phenotype [17], [18]. Most importantly however, albeit the Sost protein is structurally related to a family of Bmp antagonists, it has been shown to bind to the extracellular domain of Lrp5, thereby inhibiting the activation of Wnt signalling pathways [19]–[22].

Taken together, these results have suggested that Wnt-dependent signalling pathways are of crucial importance for osteoblast biology, which is further underscored by the fact that many mouse models with altered expression of proteins influencing Wnt binding and signal transduction display bone remodeling phenotypes [23], [24]. Among the several known modulators of Lrp5 activity, Dkk1, a member of the Dickkopf family of Wnt antagonists, appears to be particularly interesting for several reasons. First, although *Dkk1* is indispensable for embryonic head induction and limb development in mice, the postnatal analysis of *Dkk1* expression has revealed near specificity for differentiated osteoblasts [25], [26]. Second, while the homozygous deletion of *Dkk1* in mice causes embryonic lethality, the deletion of only one *Dkk1* allele results in an osteosclerotic phenotype, and the opposite is observed in transgenic mice over-expressing *Dkk1* [26], [27]. Third, although there is no report so far for an impact of *DKK1* mutations on bone mass in humans, there is hallmark evidence for an over-production of DKK1 in human cancer cells being responsible for the development of osteolytic lesions associated with metastatic bone disease [28]–[33].

Albeit Dkk1 can inhibit Wnt signalling through a direct interaction with Lrp5 or Lrp6, its antagonistic function is significantly enhanced by members of the Kremen (Krm) family, which serve as high affinity receptors for Dkk proteins [34], [35]. Whether Krm proteins solely act as antagonists of Wnt signalling is however questionable, since a positive influence on Lrp6-dependent Wnt signaling has been described for Krm2, which is possibly mediated through an interaction with Wnt signaling activators of the Rspo family [36], [37]. Here we show, that *Krm2*, unlike *Krm1*, is predominantly expressed in bone, which led us to generate transgenic mice over-expressing *Krm2* specifically in osteoblasts. These mice progressively developed an osteoporotic phenotype, which was not only caused by impaired bone formation, but also by increased bone resorption. Most importantly however, we observed that 24 weeks old *Krm2*-deficient mice, which do not display a phenotype at younger age [38], are characterized by a marked increase in bone formation. Taken together, these data identify Krm2 as a regulator of bone remodeling, at least in mice.

## Results

### *Krm2* Expression in Bone

**Figure 1:**
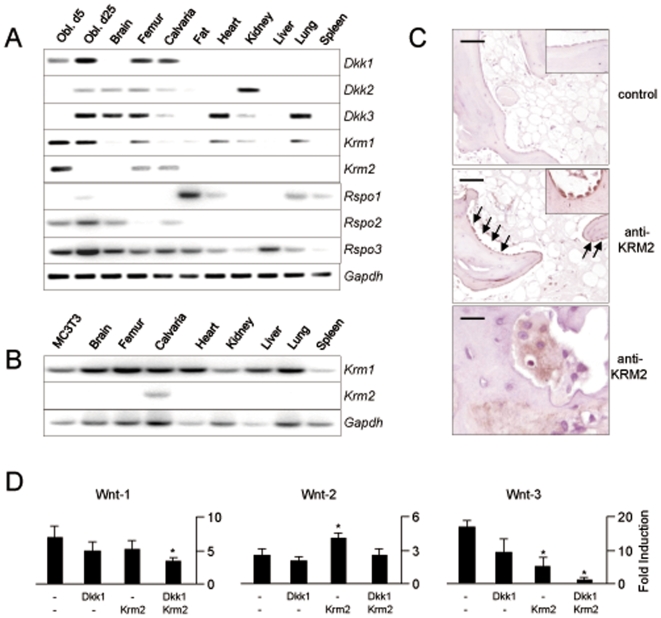
*Krm2* expression in osteoblasts. (A) RT-PCR expression analysis of *Dkk*, *Krm* and *Rspo* genes in primary osteoblasts (Obl. d5, non-mineralized, Obl. d25, mineralized) and various tissues of 6 weeks old mice. (B) RT-PCR expression analysis of *Krm* genes in non-differentiated MC3T3-E1 cells and tissues of newborn mice. (C) Immunohistochemistry on human bone sections reveals that KRM2 is present on osteoblasts lining the trabecular bone surface (arrows, scale bars, 100 µm). The bottom panel shows staining of osteoclasts (scale bars, 20 µm). (D) DNA transfection in MC3T3-E1 cells using expression plasmids for Wnt1, Wnt2 or Wnt3, Dkk1 and/or Krm2 at the indicated combinations. Bars represent mean ± SD of three independent experiments (n = 9). Asterisks indicate statistically significant changes.

To uncover the potential relevance of Krm proteins in the regulation of bone remodeling, we first analyzed the expression pattern of the two known murine *Krm* genes and their potential ligands of the Dkk and Rspo family by RT-PCR using cDNA from tissues of 6 weeks old mice. Here we observed that *Krm2*, like *Dkk1*, but unlike *Krm1*, is predominantly expressed in bone, thereby suggesting a function in the regulation of osteoblasts and/or osteoclasts (Figure 1A). A similar result was obtained, when we used tissues from newborn mice, albeit we only detected *Krm2* expression in calvarial bone, but not in the femur (Figure 1B). To analyze bone expression on the protein level, we took advantage of an antibody against the human KRM2 protein. Using immunohistochemistry on human bone sections we found that KRM2 is specifically present in osteoblasts, but not in cells of the bone marrow, albeit we also observed a weak staining of bone-resorbing osteoclasts (Figure 1C).

Taken together, these findings led us to analyze the influence of Krm2 on Wnt signaling in osteoblasts, which was first done *in vitro* using the cell line MC3T3-E1, where we did not observe endogenous expression of *Krm2* (Figure 1B) and *Dkk1* (data not shown). Using DNA-transfection we observed that Dkk1 and Krm2 antagonize the activation of a Wnt-responsive *Luciferase* reporter gene, only when Wnt1 or Wnt3 expression plasmids are co-transfected, but that *Luciferase* expression is increased by Krm2, when a Wnt2 expression plasmid is used instead (Figure 1C). Based on these conflicting results, we reasoned that it is virtually impossible to analyze the role of Krm2 in osteoblasts *in vitro*. Thus, we generated transgenic mice over-expressing *Krm2* under the control of an osteoblast-specific *Col1a1* promoter fragment [39], [40] to determine, whether Krm2 has an influence on bone remodeling *in vivo*, and if so, whether it is promoting or inhibiting osteoblast-specific activites.

### Generation of *Col1a1-Krm2* Transgenic Mice

**Figure 2:**
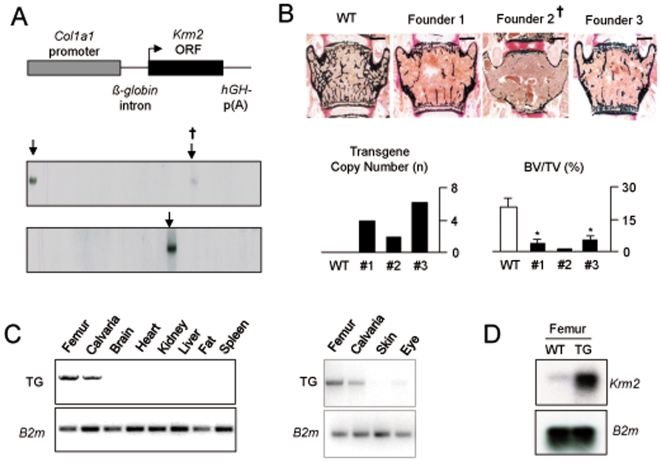
Generation of *Col1a1-Krm2* transgenic mice. (A) Schematic presentation of the construct used for osteoblast-specific over-expression of *Krm2* (top) and identification of three transgenic founder animals (arrows) by Southern Blotting (bottom), one of them dying at the age of 10 weeks. (B) Von Kossa/van Gieson staining of non-decalcified vertebral body sections from 10 weeks old female wildtype mice (n = 3), the dead founder animal (#2) and age-matched female transgenic offspring from the two other founders (n = 3, scale bars, 1 mm). The transgene copy numbers, as well as the trabecular bone volume (BV/TV, bone volume per tissue volume) is given below. (C) Transgene-specific RT-PCR expression analysis revealing expression in bone and weak expression in the eye. (D) Northern Blotting comparing *Krm2* expression in transgenic mice compared to wildtype littermates.

Three transgenic founder animals were identified by Southern Blot hybridization (Figure 2A). One of these animals died at the age of 10 weeks for unknown reasons, but its skeletal analysis demonstrated a severe reduction of bone mass, with a near absence of trabecular bone (Figure 2B). The two remaining founder animals were viable, which enabled us to establish independent transgenic lines. Since transgenic offspring of both founders also displayed a striking decrease of the trabecular bone volume at 10 weeks of age, we concluded that this phenotype is indeed the consequence of *Krm2* over-expression and not caused by an insertional inactivation of other genes. Based on these results we decided to focus on a founder line with an intermediate transgene copy number (#1), which is termed *Col1a1-Krm2* for the remainder of the manuscript (Figure 2B). Here we first performed RT-PCR to confirm the bone-specific expression of the transgene (Figure 2C), and using Northern Blot hybridization we found that the expression of *Krm2* was at least 20-fold increased in bones from transgenic animals (Figure 2D).

**Figure 3:**
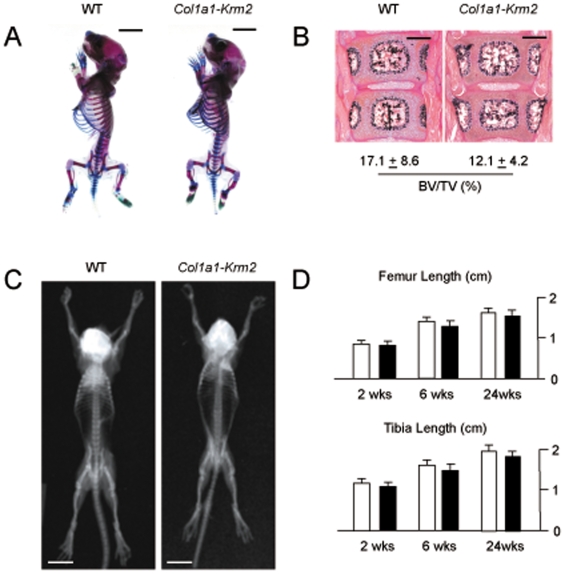
Normal skeletal patterning and growth in *Col1a1-Krm2* transgenic mice. (A) Staining of skeletons from one day old wildtype and *Col1a1-Krm2* transgenic littermates by alcian blue and alizarin red (scale bars, 5 mm). (B) Von Kossa/van Gieson staining of non-decalcified vertebral body sections from one day old wildtype and *Col1a1-Krm2* transgenic littermates (n = 4, scale bars, 500 µm). The histomorphometric quantification of the trabecular bone volume is given below. (C) Contact Xrays of 2 weeks old female wildtype and *Col1a1-Krm2* transgenic littermates (scale bars, 1 cm). (D) Determination of the femur and tibia length of female wildtype and *Col1a1-Krm2* transgenic littermates at the indicated ages. Bars represent mean ± SD (n = 6).

In order to analyze to skeletal phenotype of the *Col1a1-Krm2* mice we first stained skeletons of one day old animals with alizarin red and alcian blue, but we did not observe major defects of skeletal patterning and growth in transgenic mice compared to wildtype littermates (Figure 3A). Likewise, non-decalcified sections of the spine did not reveal a significant difference of the trabecular bone volume between newborn wildtype and transgenic mice (Figure 3B). We therefore continued our study with the skeletal analysis of older mice and first performed contact radiography, where we failed to detect significant differences of skeletal growth at 2 weeks of age (Figure 3C) and thereafter (Figure 3D).

### Severe Osteoporosis in *Col1a1-Krm2* Transgenic Mice

**Figure 4:**
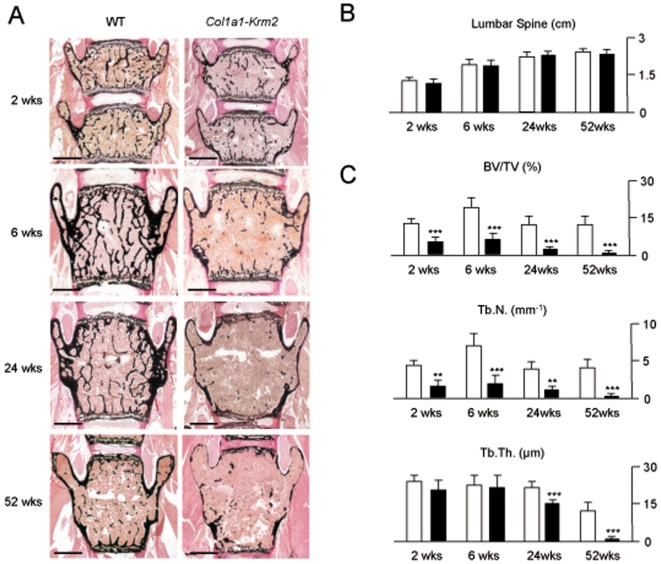
Decreased trabecular bone mass in *Col1a1-Krm2* transgenic mice. (A) Von Kossa/van Gieson staining of non-decalcified vertebral body sections from female wildtype and *Col1a1-Krm2* transgenic littermates at the indicated ages (scale bars, 1 mm). (B) Determination of the lumbar spine length. (C) Histomorphometric quantification of the trabecular bone volume, trabecular number (Tb.N.) and trabecular thickness (Tb.Th.). All bars represent mean ± SD (n = 6). Asterisks indicate statistically significant differences.

When we performed non-decalcified histology of vertebral body sections, we observed that female *Col1a1-Krm2* transgenic mice progressively develop severe osteoporosis (Figure 4A). Albeit the length of the lumbar spine was not reduced in transgenic mice (Figure 4B), our histomorphometric quantification revealed a decreased trabecular bone volume and trabecular number in *Col1a1-Krm2* mice at all ages, and a decreased trabecular thickness at 24 weeks and 52 week of age (Figure 4B). The same phenotype was observed in male transgenic mice, where we also found a more than 4-fold reduction of the trabecular bone volume compared to wildtype littermates at 24 weeks of age (data not shown).

**Figure 5:**
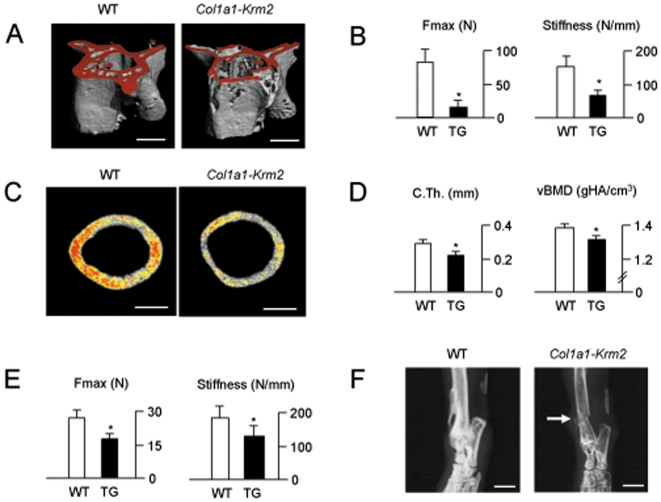
Decreased biomechanical stability of bones from *Col1a1-Krm2* transgenic mice. (A) µCT scanning of the vertebral bodies L6 from 24 weeks old female wildtype and *Col1a1-Krm2* transgenic littermates (scale bars, 1 mm). (B) Microcompression testing revealed reduced biomechanical stability (Fmax, maximal force until bone failure). Bars represent mean ± SD (n = 6). Asterisks indicate statistically significant differences. (C) µCT scanning of the femora showing reduced cortical thickness and impaired mineralization (scale bars, 500 µm). (D) Cortical thickness (C.Th.) and bone mineral density (vBMD) are decreased in *Col1a1-Krm2* transgenic mice compared to wildtype littermates. Bars represent mean ± SD (n = 6). Asterisks indicate statistically significant differences. (E) Three-point bending assays reveal reduced biomechanical competence of transgenic femora. Bars represent mean ± SD (n = 12). Asterisks indicate statistically significant differences. (F) Xray analysis of a 30 weeks old male *Col1a1-Krm2* transgenic mouse with a spontaneous tibia fracture (arrow) compared to a non-transgenic littermate (scale bars, 2 mm).

Following µCT scanning of the vertebral bodies L6 (Figure 5A), where we observed decreased trabecular bone mass, but normal cortical thickness in *Col1a1-Krm2* mice (data not shown), we performed microcompression testing and found that the biomechanical stability of the vertebral bodies is largely reduced in *Col1a1-Krm2* mice, when compared to wildtype littermates (Figure 4B). Cross-sectional µCT scans of the femora (Figure 4C) further revealed reduced cortical thickness and bone mineral content in *Col1a1-Krm2* mice (Figure 4D), again accompanied by reduced biomechanical competence in three-point-bending assays (Figure 4E). In line with these findings, 2 out of 8 transgenic mice older than 24 weeks (one male and one female) had to be sacrificed due to spontaneous fractures, thereby demonstrating increased bone fragility also *in vivo* (Figure 4F). Taken together, these analyses revealed that *Krm2* expression in osteoblasts has a negative impact on bone integrity, which led us to perform histomorphometry to determine, whether the severe osteoporosis in *Col1a1-Krm2* mice is caused by impaired bone formation and/or bone resorption.

### *Krm2* Over-expression in Osteoblasts Impairs Bone Formation

**Figure 6:**
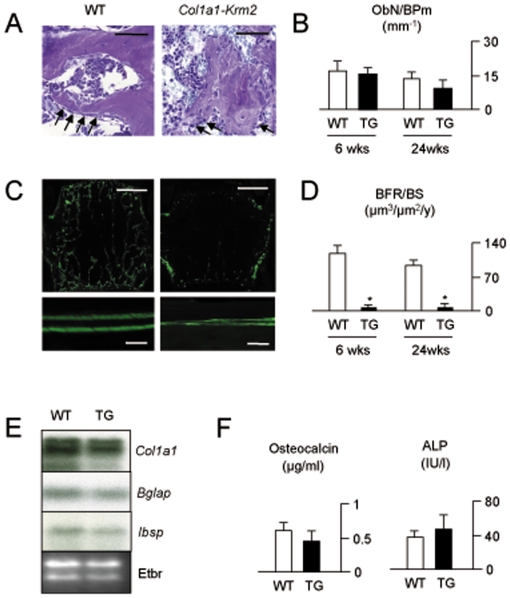
Impaired bone formation in *Col1a1-Krm2* transgenic mice. (A) Toluidine blue staining of non-decalcified vertebral body sections from 6 weeks old female wildtype and *Col1a1-Krm2* transgenic littermates revealing that the normal appearance of cuboidal osteoblasts (arrows) covering trabecular bone surfaces is only observed in wildtype controls (scale bars, 50 µm). (B) Histomorphometric quantification showing that the number of osteoblasts (ObN/BPm, osteoblast number per bone perimeter) is not significantly decreased in sections from transgenic mice. (C) Fluorescent micrographs showing that overall calcein labeling is reduced in vertebral bodies of 6 weeks old female *Col1a1-Krm2* transgenic mice (top, scale bars, 1 mm), as is the distance between the labeled surfaces at endosteal bone surfaces of the tibia (bottom, scale bars, 20 µm). (D) Histomorphometric quantification of the bone formation rate (BFR/BS, bone formation rate per bone surface) in female wildtype and *Col1a1-Krm2* transgenic littermates. (E) Northern Blot expression analysis for *Col1a1*, *Bglap* and *Ibsp* using femur RNA of 6 weeks old female wildtype and transgenic mice. (F) Serum levels of osteocalcin and activities of alkaline phosphatase in 6 weeks old female wildtype and transgenic mice. All bars represent mean ± SD (n = 6). Asterisks indicate statistically significant differences.

Although the histomorphometric quantification did not reveal a significant difference of osteoblast numbers between wildtype and transgenic mice, there was a striking effect of *Krm2* over-expression on osteoblast maturation and bone matrix deposition (Figure 6A,B). In fact, none of the osteoblasts observed in sections from transgenic mice displayed the regular morphology, and there was a non-homogenous staining of mineralized bone compared to sections from wildtype littermates. Likewise, when we looked at the sections by fluorescence microscopy we observed that the number of calcein-labelled surfaces, as well as the distance between calcein labelling fronts was dramatically reduced in *Col1a1-Krm2* mice (Figure 6C). The quantification of these findings revealed that the bone formation rate was nearly undetectable in *Col1a1-Krm2* mice at 6 and 24 weeks of age (Figure 6D). Interestingly, this near abolishment of bone formation was not accompanied by changes in the expression of well-established osteoblast differentiation markers, such as *Col1a1*, *Bglap* or *Ibsp* (Figure 6E), which is further underscored by the finding that serum osteocalcin levels and alkaline phosphatase activities were not significantly decreased in 6 weeks old female *Col1a1-Krm2* mice (Figure 6F).

### Impaired Differentiation and Decreased Opg Production in Primary Osteoblasts from *Col1a1-Krm2* Transgenic Mice

**Figure 7:**
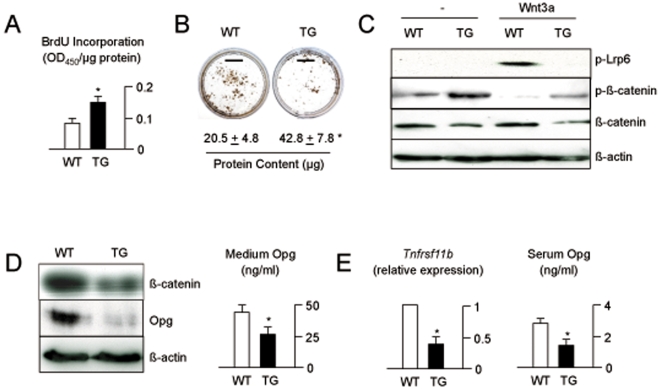
Cell-autonomous defect of *Col1a1-Krm2* transgenic osteoblasts. (A) BrdU incorporation assays revealed a higher proliferation rate in primary calvarial osteoblast cultures from transgenic mice after 2 days of differentiation. (B) Von Kossa staining performed at 10 days of differentiation reveals reduced mineralization of osteoblasts from *Col1a1-Krm2* mice (scale bars, 1 cm), despite higher protein content (given below). Values represent mean ± SD (n = 3). Asterisks indicate statistically significant differences. (C) Western Blot analysis of canonical Wnt signaling using primary osteoblasts from wildtype and transgenic mice following stimulation with Wnt3a for 30 minutes. (D) Western Blot analysis (left) and ELISA (right) showing decreased Opg levels in cellular extracts and conditioned medium of osteoblasts from *Col1a1-Krm2* mice. (E) Quantitative RT-PCR (left) and ELISA (right) demonstrating that the reduced expression of *Tnfrsf11b* in bones of 6 weeks old *Col1a1-Krm2* mice results in decreased Opg serum levels. All bars represent mean ± SD (n = 4). Asterisks indicate statistically significant differences.

Taken together, these findings led us to analyze the molecular differences between wildtype and transgenic osteoblasts, which were isolated from calvariae and differentiated *ex vivo*. Here we observed that, although their proliferation rate was significantly increased (Figure 7A), cultures from transgenic mice displayed the expected decrease of extracellular matrix mineralization after 10 days of differentiation (Figure 7B). Moreover, when we stimulated primary osteoblasts with Wnt3a we observed that the phosphorylation of Lrp6 was blunted in cultures from transgenic mice (Figure 7C). Likewise, the Wnt3a-dependent activation of canonical Wnt signaling, assessed by decreased phosphorylation of ß-catenin, was markedly reduced in transgenic cultures, as were the levels of total ß-catenin. Since the expression of *Tnfrsf11b*, the gene encoding the osteoclast-inhibitory factor Opg, is known to be induced by canonical Wnt signaling in osteoblasts [41], we next determined Opg production in osteoblasts from *Col1a1-Krm2* mice and observed that it was reduced compared to wildtype cultures, both by Western Blotting using cellular extracts and by ELISA using conditioned medium (Figure 7D). Similar results were obtained *in vivo*, where we found decreased expression of *Tnfrsf11b* by quantitative RT-PCR, as well as lower Opg serum concentrations in 6 weeks old female *Col1a1-Krm2* mice (Figure 7E).

### Increased Bone Resorption and Osteolytic Lesions in *Col1a1-Krm2* Mice

**Figure 8:**
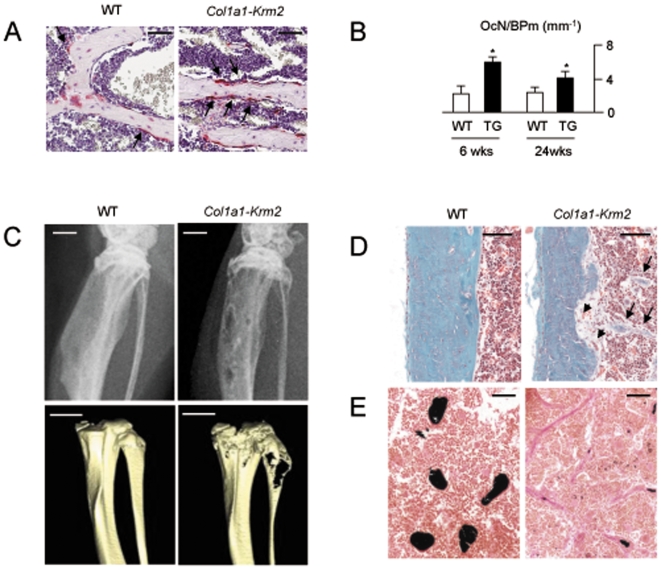
Increased bone resorption in *Col1a1-Krm2* transgenic mice. (A) TRAP activity staining for osteoclasts (arrows) in decalcified vertebral body sections from 6 weeks old female wildtype and *Col1a1-Krm2* transgenic mice (scale bars, 50 µm). (B) Histomorphometric quantification confirmed the increased number of osteoclasts (OcN/BPm, osteoclast number per bone perimeter) in transgenic mice. Bars represent mean ± SD (n = 6). Asterisks indicate statistically significant differences. (C) Xray analysis (top, scale bars, 1 mm) and µCT scanning (bottom, scale bars, 2 mm) demonstrating the presence of osteolytic lesions in 52 weeks old female *Col1a1-Krm2* mice. (D) Goldner staining of the tibia showing osteoclasts at sites of cortical bone erosion (arrowheads), but also inappropriate bone formation in the marrow cavity of 52 weeks old female transgenic mice (arrows, scale bars, 100 µm). (E) Von Kossa/van Gieson staining reveals that the inappropriate bone formation in 52 weeks old female *Col1a1-Krm2* mice is associated with an accumulation of non-mineralized osteoid (stained in red, scale bars, 100 µm).

Based on these findings, we next analyzed the influence of *Krm2* over-expression on bone resorption. Using TRAP activity staining we found increased numbers of osteoclasts in spine sections from transgenic mice (Figure 8A), which was subsequently confirmed by histomorphometry (Figure 8B). Another important observation was made, when we analyzed the *Col1a1-Krm2* mice at the age of 52 weeks, where we found osteolytic lesions, that were especially pronounced in the lower extremities (Figure 8C) and histologically confirmed to be caused by a local activation of bone resorption (Figure 8D). In addition, our histological analysis revealed several sites of inappropriate *de novo* bone formation within the marrow cavity of *Col1a1-Krm2* mice (Figure 8E). Taken together, these results demonstrated that Krm2, at least in mice, is a potent inhibitor of bone formation, with an additional influence on bone resorption, thereby underscoring the importance of Wnt signaling in osteoblasts for both arms of bone remodeling.

### Identification of differentially expressed genes in *Col1a1-Krm2* transgenic osteoblasts

**Figure 9:**
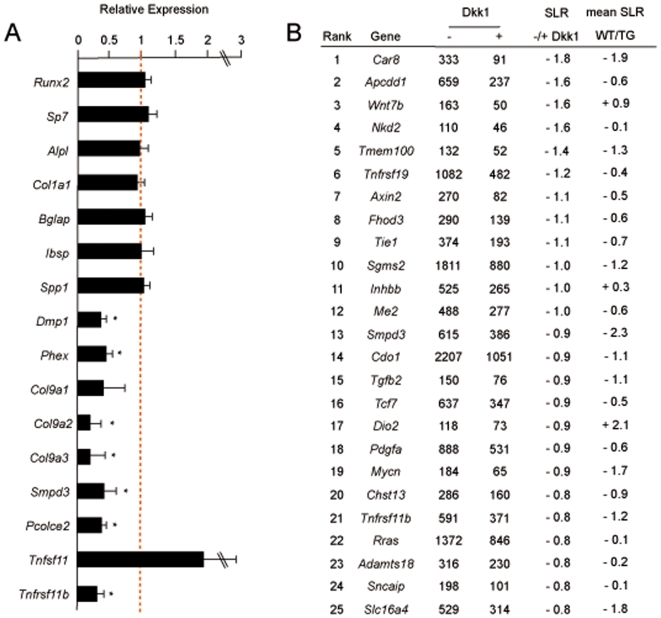
Differentially expressed genes in *Col1a1-Krm2* transgenic osteoblasts. (A) Affymetrix Gene Chip hybridization demonstrates that several well-established osteoblast differentiation markers are expressed at similar levels in osteoblasts from wildtype (mean values indicated by the dotted red line) and transgenic mice, while other genes are expressed at lower levels in the latter ones. Bars represent mean ± SD (n = 3). Asterisks indicate statistically significant differences between the relative signal intensities in wildtype and transgenic samples. (B) Affymetrix Gene Chip hybridization of wildtype osteoblasts following treatment with Dkk1 for 6 hours at day 10 of differentiation (n = 1). Shown are the Affymetrix signal intensities and the signal log ratios (SLR) for the 25 genes displaying the strongest negative regulation by Dkk1. The mean signal ratios of the Gene Chip comparison between wildtype and *Col1a1-Krm2* transgenic osteoblasts are given on the right.

To identify genes with a possible involvement in the cell-autonomous defect of bone formation caused by the *Krm2* over-expression, we next performed Affymetrix Gene Chip hybridization, where we applied samples from three independently isolated wildtype and transgenic osteoblast cultures at day 10 of differentiation (Figure 9A). While we did not observe altered expression of several well-established osteoblast differentiation markers, such as *Runx2*, *Sp7*, *Alpl*, *Col1a1*, *Bglap*, *Ibsp* and *Spp1* in transgenic cultures, our analysis revealed significantly reduced expression of specific genes, albeit only few of them can potentially explain the severe impairment of bone formation caused by *Krm2* over-expression. These included *Dmp1* [42], *Phex* [43], the three genes encoding type-IX-collagen [44], *Smpd3* [45], and *Pcolce2*, the latter one encoding an enhancer of procollagen processing [46]. In addition, the expression of *Tnfsf11*, encoding the osteoclast differentiation factor Rankl, was higher in transgenic osteoblasts, whereas the expression of *Tnfrsf11b* was decreased.

To address the question, whether these genes are direct targets of Dkk1, we compared these results to another Affymetrix Gene Chip hybridization experiment, where we treated wildtype osteoblasts with Dkk1 for 6 hours. In line with the findings in *Col1a1-Krm2* osteoblasts we did not observe an effect of Dkk1 on the expression of *Runx2*, *Sp7*, *Alpl*, *Col1a1*, *Bglap*, *Ibsp* and *Spp1* (data not shown), but we did observe a reduced expression of *Smpd3* and *Tnfrsf11b* following Dkk1 treatment (Figure 9B). Moreover, nearly all of the 25 genes showing the strongest down-regulation by Dkk1 were also expressed at lower levels in *Col1a1-Krm2* osteoblasts. In contrast however, Dkk1 administration did not have an immediate effect on the expression of *Dmp1*, *Phex*, *Col9a1-3* and *Pcolce2* (data not shown) thereby suggesting that some of the molecular differences between wildtype and *Col1a1-Krm2* osteoblasts may be caused either indirectly or independent of Dkk1.

### High Bone Mass in Mice Lacking *Krm2*

**Figure 10:**
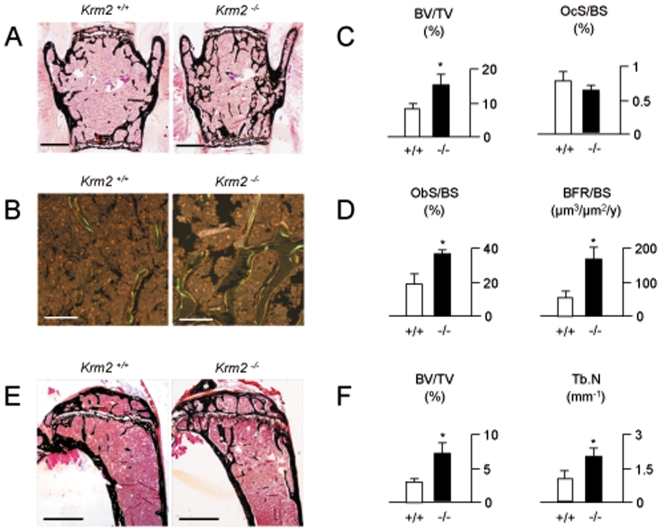
Increased bone formation in *Krm2*-deficient mice. (A) Von Kossa/van Gieson staining of non-decalcified vertebral body sections from 24 weeks old female wildtype (*Krm2^+/+^*) and *Krm2*-deficient (*Krm2^−/−^*) mice (scale bars, 1 mm). (B) Fluorescent micrographs showing a higher number of calcein-labelled surfaces in *Krm2*-deficient vertebral bodies (scale bars, 200 µm). (C) Histomorphometric quantification of the trabecular bone volume and osteoclast surface per bone surface (OcS/BS). (D) Histomorphometric quantification of the osteoblast surface per bone surface (ObS/BS) and the bone formation rate. (E) Von Kossa/van Gieson staining of non-decalcified tibia sections from 24 weeks old female wildtype and *Krm2*-deficient mice (scale bars, 1 mm). (F) Histomorphometric quantification of the trabecular bone volume and the trabecular number. All bars represent mean ± SD (n = 6). Asterisks indicate statistically significant differences.

Given the potential importance of these findings for the treatment of bone loss disorders, we finally addressed the question, whether the observed effects of Krm2 are physiologically relevant. To achieve this goal, we analyzed vertebral bodies of 24 weeks old *Krm2*-deficient mice, since the previous analysis of tibia sections at the age of 12 weeks did not reveal a significant increase of bone mass compared to wildtype littermates [38]. Here we found that 24 weeks old *Krm2*-deficient mice displayed a significantly increased trabecular bone mass (Figure 10A), together with an increased number of single- and double-labeled bone surfaces following dual calcein injection (Figure 10B). Using histomorphometry we were able to confirm the increase of the trabecular bone volume, but we failed to detect a defect of osteoclast formation in *Krm2*-deficient mice at 24 weeks of age (Figure 10C). In contrast, the osteoblast surface was significantly higher in *Krm2*-deficient mice, and most importantly, the bone formation rate was more than three-fold increased compared to wildtype littermates (Figure 10D). Moreover, unlike it was the case at the age of 12 weeks, 24 weeks old animals *Krm2*-deficient mice also displayed an increased trabecular bone volume in the tibia (Figure 10E,F). Taken together, these results demonstrate that Krm2, at least in mice, is an endogenous inhibitor of bone formation.

## Discussion

Although osteoporosis is one of the most prevalent diseases in the aged population, the options for its treatment are still limited [47]. This is especially true for agents stimulating the activity of osteoblasts, which explains why the discovery of LRP5 and SOST as regulators of bone formation in humans was considered to be of hallmark clinical importance [5]–[9]. Here we show, that the transmembrane protein Krm2, which serves as a receptor for Wnt antagonists of the Dkk family [34], is another physiological regulator of bone formation, at least in mice. In fact, *Col1a1-Krm2* transgenic mice display a severe osteoporotic phenotype, resulting in reduced biomechanical stability of both, vertebral bodies and long bones. This can be primarily explained by a decreased trabecular bone mass, whereas cortical thickness was only found affected in femora, but not in the spine, which might be explained by different strain distributions between the two skeletal elements. Most importantly however, *Krm2*-deficient mice also display a high trabecular bone mass phenotype at 24 weeks of age, which is especially relevant given the absence of obvious abnormalities outside the skeleton. Based on these arguments, it appears that Krm2 has a specific function in bone remodeling, thereby raising the possibility that KRM2 might serve as an ideal target for osteoanabolic therapy, if it exerts a similar function in humans. In this regard, it is important to state that an over-expression of *Krm2* in osteoblasts also causes an activation of bone resorption, which implies that a putative KRM2 antagonist might have a possible influence on both arms of bone remodeling.

Albeit our results obtained in two genetically modified mouse models confirm that an antagonism of Wnt signaling in osteoblasts has a negative effect on bone formation, they were not necessarily expected, based on several findings published by others. First, while mice lacking one allele of *Dkk1* display the expected high bone mass phenotype, *Dkk2*-deficient mice are characterized by reduced bone formation and osteopenia, thereby demonstrating that these two Krm2 ligands do not mediate the same effects on osteoblasts *in vivo* [48]. Second, while bone mass is unaffected in mice lacking *Lrp5* specifically in osteoblasts, a gut-specific deletion of the *Lrp5* gene resulted in decreased bone formation as a consequence of increased serotonin production, thus implying that Lrp5 does not control bone formation in a cell-autonomous manner [49]. Third, mice harbouring an osteoblast-specific inactivation of ß-catenin, the major intracellular mediator of Wnt signalling, displayed normal bone formation, but increased bone resorption, which was molecularly explained by reduced production of the osteoclast-inhibitory factor Opg [41]. Taken together, these two latter findings have challenged the concept that Wnt signalling in osteoblasts is a key pathway for the regulation of bone formation, which was further underscored by the finding that the osteopenia of *rs/rs* mice, carrying a hypomorphic mutation of the Wnt co-receptor Lrp6, is caused by increased bone resorption [50].

In this regard, it was not only an important, but also an unexpected observation, that the osteoblast-specific antagonism of Wnt signalling achieved through transgenic over-expression of *Krm2* has such a tremendous impact on bone formation, thereby causing an osteoporotic phenotype, whose severity exceeds the one observed in *Lrp5*-deficient mice [11]. In fact, our finding that bone formation was nearly abolished in the *Col1a1-Krm2* transgenic mice underscores the importance of intact Wnt signalling for the endogenous regulation of osteoblast differentation and function. Another interesting and unexpected observation was that the severe impairment of bone formation in *Col1a1-Krm2* mice occurred despite nearly normal expression levels of several well-established osteoblast differentiation markers, which was confirmed by a genome-wide comparative expression analysis using three independently isolated osteoblast cultures from wildtype and *Col1a1-Krm2* transgenic mice. Since we did observe a cell-autonomous defect of matrix mineralization in primary osteoblasts from *Col1a1-Krm2* mice however, we were further able to identify specific genes, whose reduced expression can possibly explain the severe impairment of bone formation in *Col1a1-Krm2* mice. These included *Dmp1* and *Phex*, two genes required for bone matrix mineralization and phosphate homeostasis (41,42), but also the three genes encoding type IX collagen, which is potentially important, since *Col9a1*-deficient mice have recently been described to display an osteoporotic phenotype [44]. However, since we only observed a moderate increase in the volume of non-mineralized osteoid in *Col1a1-Krm2* mice, together with normal serum phosphate levels (data not shown), and since the osteoporotic phenotype of *Col9a1^+/−^* mice is rather caused by increased bone resorption [44], we believe that the reduced expression of other genes is more likely to explain the near absence of bone formation in *Col1a1-Krm2* mice.

Of particular interest in this regard is *Smpd3*, encoding one member of the sphingomyelin phosphodiesterase family, cleaving sphingomyelin into ceramide [51]. Although the precise mechanism of its action in osteoblasts remains to be established, a deletion within the *Smpd3* gene has been found in a chemically induced mutant mouse model termed *fragilis ossium (fro)* [45]. The *fro/fro* mice have been described as a model of osteogenesis imperfecta (OI), a genetic disease of impaired bone matrix deposition and increased fracture risk [52]. However, unlike what is the case in the vast majority of OI cases, *fro/fro* mice do not display any detectable collagen synthesis defect, thereby suggesting that sphingomyelin degradation may be a key factor regulating formation and mineralization of the bone matrix, independent of collagen production [53], [54]. In this regard it is also interesting that another gene with significantly reduced expression in osteoblasts from *Col1a1-Krm2* mice, namely *Pcolce2*, has been described to serve a function as an enhancer of C-terminal procollagen processing [46]. Whether this function is also relevant for the posttranslational modification of type I collagen in osteoblasts remains to be established, since mice lacking *Pcolce2* have not been analyzed for their skeletal phenotype so far [55].

Given the fact, that the phenotype of *Col1a1-Krm2* mice is apparently more severe than the phenotype of *Col1a1-Dkk1* transgenic mice reported in the literature [26], it was also important to compare the genome-wide expression analysis of wildtype and *Col1a1-Krm2* transgenic osteoblasts to another Gene Chip hybridization experiment, where we have treated wildtype osteoblasts with Dkk1 for 6 hours. Interestingly, the data obtained here confirmed that the majority of genes being repressed following Dkk1 administration were also expressed at lower levels in osteoblasts from *Col1a1-Krm2* mice. In contrast however, we did not find an influence of Dkk1 on the expression of *Dmp1*, *Phex*, *Pcolce2* and the genes encoding type IX collagen. This suggests that Krm2 might also exert Dkk1-independent functions, and that future experiments should aim at the identification of other signaling pathways that might be affected by Krm2. In addition, it might be worthwhile to analyze the expression of *Smpd3* and *Tnfrsf11b* in osteoblasts from *Col1a1-Dkk1* mice. In this regard, it is especially surprising that the *Col1a1-Dkk1* mice do not display increased bone resorption, since a negative influence of Dkk1 on Opg production has also been reported by others [56], [57].

Regardless of the precise mechanism underlying the effects of *Krm2* on bone remodeling however, we believe that the most important question is whether the deduced functions of Krm2 are also physiologically relevant. The potential role of the two known *Krm* genes in bone remodeling has recently been addressed through the analysis of the respective mouse deficiency models [38]. Not necessarily expected, both mouse models were viable and fertile and neither displayed obvious abnormalities, nor premature mortality. The same was the case for mice lacking both *Krm* genes, thereby demonstrating that Krm-dependent signalling pathways are dispensable for most developmental and physiological processes. A histomorphometric analysis of bone remodeling, performed in tibia sections of 12 weeks old *Krm1-* and *Krm2*-deficient mice, revealed no significant difference compared to wildtype littermates. However, the combined deficiency of both *Krm* genes resulted in increased bone formation and osteosclerosis, thereby suggesting a physiological influence of Krm1 and Krm2 on bone formation with functional redundancy.

Based on our findings obtained in the *Col1a1-Krm2* transgenic mice we have now expanded the analysis of *Krm2*-deficient mice to 24 weeks of age and observed a marked increase of the trabecular bone volume compared to wildtype littermates, which is caused by a more than three-fold increase of bone formation. In contrast to the results obtained in the transgenic animals however, we did not observe a difference in osteoclast number, eroded surface or in the serum concentrations of carboxyterminal collagen crosslinks (data not shown) between wildtype and *Krm2*-deficient littermates. It is possible that these changes would appear with time, since our own experience, not only with the *Col1a1-Krm2* mice, but also with *Calca*- or *Cckbr*-deficient mice for instance, raises the possibility, that severe bone resorption phenotypes, such as osteolyses, rather develop in aged mice [58], [59]. In this regard it will be interesting to study the skeletal phenotype of *Krm2*-deficient mice being older than one year, and it may be worthwhile to analyze the possibility that these mice are protected from tumor-induced osteolytic lesions. Another possibility explaining the absence of a bone resorption phenotype in 24 weeks old *Krm2*-deficient mice would be that the regulation of osteoclastogenesis is compensated by Krm1. To address this question it will be important to analyze 24 weeks old mice lacking either *Krm1* alone, or both murine *Krm* genes in future experiments. Regardless of the outcome from this analysis however, the results presented in this manuscript provide important and novel *in vivo* evidence for a specific role of Krm2 in the regulation of osteoblast differentiation and activity, at least in mice.

## Materials and Methods

### Expression Analysis

RNA from mouse tissues, primary osteoblasts and MC3T3-E1 cells was isolated using the Trizol reagent (Invitrogen) and reverse transcribed using the Cloned AMV First-Strand cDNA synthesis kit (Invitrogen). PCR was performed with gene-specific primers for *Dkk1* (5′-CCA CAC CTG CCA GAG ACA CTA AAC-3′and 5′-GGG GAG TTC CAT CAA GAA ACA AAG-3′), *Dkk2* (5′-CCT ACT CTT CCA AAG CCA GAC TCC-3′and 5′-TGA CAA TCT GAA GGA AAT GCC-3′), *Dkk3* (5′-TAG GCG GAG AGG AGG AGA TTT AGG-3′ and 5′-GGT TAC ATT TTG CCA AGT CCA CG-3′), *Krm1* (5′-AAC GAG ACT TTC CAG CAT CCG-3′and 5′-TCC ATC CCA GCA AAC TTG AAT C-3′), *Krm2* (5′-TGG GTT CCT ACA GAA GTT ATG CG-3′and 5′-CGT CCA AGG CAC CAT CTC TTT G-3′), *Rspo1* (5′-ACC TGG ATA CTT TGA TGC CCG-3′and 5′-CGC TCA TTT CAC ATT GTG CAG′), *Rspo2* (5′-GAA GTT GGT CAT TGG AGC GAA-3′and 5′-TGC CTT TGG TGT TCT CTT TCC T′), *Rspo3* (5′-AAA GTG CCT TGA CAG TTG CGC-3′ and 5′-TCC TCG CTC TCC CTT TGA ACA C-3)and *Gapdh* (5′-GAC ATC AAG AAG GTG GTG AAG CAG-3′ and 5′-CTC CTG TTA TTA TGG GGG TCT GG-3′), and the identity of amplified fragments was verified by automatic sequencing. Northern Blot analysis was carried out according to standard protocols [60]. For the genome-wide expression analysis, RNA was subjected to hybridization of Affymetrix Gene Chips (MG 430 2.0) according to the manufacturer's protocol. Absolute and comparison analyses were performed with Affymetrix MAS algorithm using default parameters. Quantitative RT-PCR analysis was performed using a StepOnePlus system, predesigned TaqMan gene expression assays, and TaqMan gene expression mastermix (Applied Biosystems). *Gapdh* and *B2m* expression was used as internal controls. Relative quantification was performed according to the ΔΔC_T_ method, and results were expressed in the linear form using the formula 2^−ΔΔC^T.

### Human Bone Biopsies

A collection of biopsies from healthy individuals and from patients with various bone disorders has been established at the Hamburg University over the last decades [61]. Immunohistochemistry was performed on decalcified sections from skeletal-intact donors using a polyclonal antibody against human KRM2 (Sigma, #HPA003223) following standard protocols.

### DNA Transfection

The three different *Wnt* expression plasmids were kindly provided by Dr. J. Kitajewski (New York, USA), the *Krm2* expression plasmid has been described previously [34], and the *Dkk1* expression plasmid was constructed by placing the full-length cDNA into the vector pCMV-Tag4 (Stratagene). In all experiments the Wnt-responsive reporter plasmid TOPflash (Upstate, #21-170) was co-transfected with a CMV-driven ß-Galactosidase reporter plasmid (kindly provided by Dr. P. Ducy, New York, USA) to normalize for transfection efficiency. DNA-cotransfection was performed in MC3T3-E1 cells (ATCC, #CRL-2593) using calcium phosphate transfection, and the activities of Luciferase and ß-Galactosidase were measured two days later as described [62].

### Mice

For the generation of *Col1a1-Krm2* transgenic mice the ORF encoding Krm2 was placed under the control of an osteoblast-specific 2.3kb *Col1a1* promoter fragment (kindly provided by Dr. B. de Crombrugghe, Houston, USA). Pronucleus injection into fertilized oocytes was performed according to standard protocols. Genotyping of the offspring was performed by Southern Blotting following digestion with Kpn I using the 3′-UTR of the *hGH* gene as a probe, or by PCR using primers located within the ß-*globin* intron and the *Krm2* cDNA. To determine the transgene copy number genomic DNA was digested with Kpn I and probed with a Kpn I-fragment of the *Krm2* cDNA, which detected a transgenic fragment of 888 bp and an endogenous fragment of 3.5 kb. Transgene expression was monitored by RT-PCR using primers within the *Krm2* cDNA and the 3′-UTR of the *hGH* gene. The generation of *Krm2*-deficient mice has been described previously [38]. All animal experiments were approved by the animal facility of the University Medical Center Hamburg-Eppendorf and by the “Amt für Gesundheit und Verbraucherschutz” (35/04, Org139).

### Skeletal Analysis

Before their skeletal analysis all mice received two injections of calcein (9 and 2 days before sacrifice). After their initial analysis by contact Xray (Faxitron Xray Corp.), the vertebral bodies L2 to L5 and one tibia from each animal were dehydrated and embedded non-decalcified into methylmetacrylate for sectioning. Sections were either stained with toluidine blue, by the von Kossa/van Gieson procedure, or by Goldner staining as described [58]. µCT scanning was performed with vertebral bodies L6 and with femora using a µCT 40 device (Scanco Medical, Switzerland). Biomechanical stability was assessed by microcompression testing for vertebral bodies and by three-point bending assays for femora using a Z2.5/TN1S-device (Zwick) [59]. Staining of skeletons from newborn mice with alcian blue and alizarin red was performed as described [60].

### Quantification of Bone Remodeling

Static and cellular histomorphometry was carried out on toluidine blue-stained sections using the OsteoMeasure system (Osteometrics, Decatur, USA) following the guidelines of the American Society of Bone and Mineral Research [63]. Dynamic histomorphometry for determination of the bone formation rate was performed on two consecutive non-stained 12µm-sections. TRAP activity staining was performed on decalcified sections using napthol AS-MX phosphate (Sigma, #N-5000) and Fast Red Violet LB salt (Sigma, #F-3881) in 40 mM acetate buffer (pH5). Serum levels of Osteocalcin and Opg were determined by RIA (Immutopics, #50-1300) and ELISA (R&D Systems, #MOP00), respectively. Serum alkaline phosphatase activities were measured using p-nitrophenylphosphate as a substrate (Pointe Scientific, #A7516).

### Cell culture

Primary osteoblasts were isolated from the calvariae of 5 days old mice and differentiated *ex vivo* by the addition of ascorbic acid (50 µg/ml) and ß-glycerophosphate (10 mM). BrdU incorporation assays were performed at day 2 of differentiation using the Cell Proliferation Biotrak ELISA (GE Healthcare, #RON250), while extracellular matrix mineralization was assessed at day 10 of differentiation using von Kossa-staining [60]. To demonstrate impaired Wnt signaling serum-starved cells were treated for 30 minutes with 100 ng/ml recombinant Wnt3a (R&D systems, #1224-WN-002), and whole cell lysates were subsequently used for Western Blotting using antibodies against phospho-Lrp6 (Cell Signaling, #2568), phospho-ß-catenin (Cell Signaling, #9561), total ß-catenin (Santa Cruz, #E2808), Opg (R&D Systems, #AF459) and ß-actin (Sigma, #A2228). To identify Dkk1-regulated genes, primary osteoblasts were serum-starved over night and treated with 250 ng/ml recombinant Dkk1 (R&D Systems, #5897-DK-1010) for 6 hours before the RNA was isolated as described above.

### Statistical Analysis

Results are presented as means ± standard deviations. Statistical analysis was performed using unpaired, two-tailed Student's t test, and p-values below 0.05 were considered statistically significant.
